# Proteomic analysis of egg white heparin-binding proteins: towards the identification of natural antibacterial molecules

**DOI:** 10.1038/srep27974

**Published:** 2016-06-13

**Authors:** Nicolas Guyot, Valérie Labas, Grégoire Harichaux, Magali Chessé, Jean-Claude Poirier, Yves Nys, Sophie Réhault-Godbert

**Affiliations:** 1 INRA, UR83 Recherches Avicoles, Fonction et Régulation des Protéines de l’Oeuf, F-37380 Nouzilly, France; 2 INRA, UMR85 Physiologie de la Reproduction et des Comportements-CNRS UMR 7247-Université François Rabelais-Institut Français du Cheval et de l’Equitation, Plate-forme d’Analyse Intégrative des Biomolécules (PAIB), Laboratoire de Spectrométrie de Masse, F-37380 Nouzilly, France

## Abstract

The chicken egg resists most environmental microbes suggesting that it potentially contains efficient antimicrobial molecules. Considering that some heparin-binding proteins in mammals are antibacterial, we investigated the presence and the antimicrobial activity of heparin-binding proteins from chicken egg white. Mass spectrometry analysis of the proteins recovered after heparin-affinity chromatography, revealed 20 proteins, including known antimicrobial proteins (avidin, lysozyme, TENP, ovalbumin-related protein X and avian bêta-defensin 11). The antibacterial activity of three new egg candidates (vitelline membrane outer layer protein 1, beta-microseminoprotein-like (LOC101750704) and pleiotrophin) was demonstrated against *Listeria monocytogenes* and/or *Salmonella enterica* Enteritidis. We showed that all these molecules share the property to inhibit bacterial growth through their heparin-binding domains. However, vitelline membrane outer layer 1 has additional specific structural features that can contribute to its antimicrobial potential. Moreover, we identified potential supplementary effectors of innate immunity including mucin 5B, E-selectin ligand 1, whey acidic protein 3, peptidyl prolyl isomerase B and retinoic acid receptor responder protein 2. These data support the concept of using heparin affinity combined to mass spectrometry to obtain an overview of the various effectors of innate immunity composing biological milieus, and to identify novel antimicrobial candidates of interest in the race for alternatives to antibiotics.

Antibiotics are the most common molecules used to treat bacterial infections. However, in the past few years, their use has been questioned due to the emergence of resistant bacterial strains. Consequently, many efforts are currently conducted to identify new potential molecules that might be used as alternatives or in combination to antibiotherapy. Amongst these, antimicrobial peptides, which are major components of innate immunity of most living organisms, retain much interest^1^,^2^. Interestingly, several heparin-binding proteins and peptides have been shown to exhibit antimicrobial activities^3^,^4^,^5^. In fact, the characterization of new natural antimicrobial proteins and peptides with heparin (negatively charged glycosaminoglycan) affinity represent a promising approach to provide templates for designing new therapeutic agents.

In oviparous, the embryonic development occurs in the egg, which is a closed chamber containing all protective systems and nutrients required for the proper development of an embryo. Indeed, after oviposition, there is no more possibility of exchange with the mother to fulfill embryo needs. The innate immune system in avian eggs is the only one to be functional during embryonic development until hatch, while the components of the adaptive immune system are absent up to that time point. Therefore, avian eggs constitute a potential rich source of antimicrobials and other molecules with many biological activities^6^. The internal part of eggs is usually germ-free at oviposition. Remarkably, the unfertilized chicken egg can remain sterile for at least 3 weeks at room temperature, which implies that the egg natural defenses are highly efficient to prevent entry and development of pathogens. Among these, the eggshell constitutes a physical barrier against microbes while the egg white and the vitelline membrane (acellular protein membrane surrounding the egg yolk) contain powerful antimicrobial agents such as lysozyme and ovotransferrin^6^. It is noteworthy that egg white lysozyme actually constitutes one of the active principles of many medicines to treat non-severe infections. It is also routinely used as a food preservative^7^. Many minor proteins of egg white have been recently revealed by proteomics^8^,^9^ and preliminary analysis of their sequences using bioinformatic tools allowed to predict the antimicrobial properties of about 90 of these^10^.

In this report, we investigated the antimicrobial potential of the components of the heparin-bound fraction of chicken egg white. The results presented in this publication might be of major interest for industrial and pharmaceutical applications as the strategy can be easily applied to other biological fluids including egg yolk, eggshell waste but also milk, honey, saliva, colostrum or other body fluids, to identify new natural antimicrobial molecules.

## Results

### Identification of heparin-binding proteins from egg white

**Figure 1:**
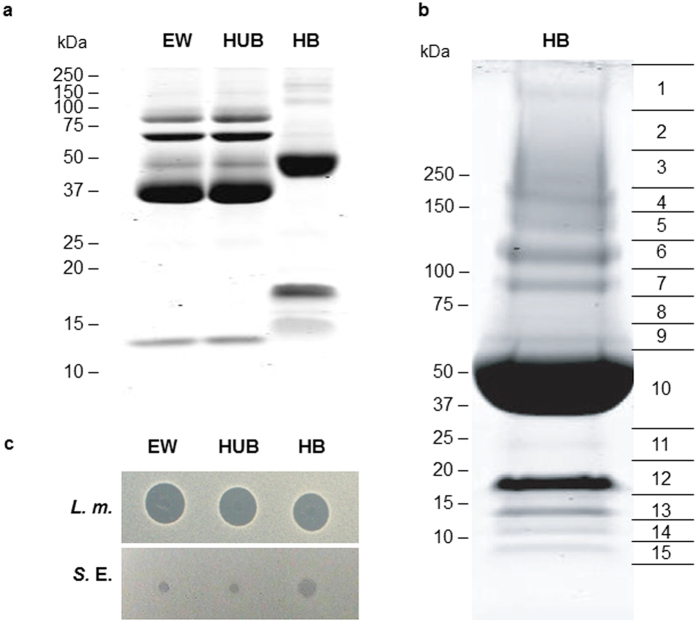
Analysis of egg white proteins before and after fractionation by heparin-affinity chromatography. (**a**) 12.5% SDS-PAGE analysis of total egg white proteins (EW), heparin-unbound fraction of egg white (HUB) and heparin-bound fraction of egg white (HB). (**b**) 4–20% SDS-PAGE analysis of egg white proteins (250 μg) purified by heparin-Sepharose affinity chromatography (HB) under non-reducing conditions. Horizontal lanes and numbers indicate the position of gel slices prepared for in-gel digestion as the initial step for mass spectrometry analysis. (**c**) Antibacterial activity of EW fractions (5 μL at 500 μg/mL) against *Listeria monocytogenes* (*L.m.*) and *Salmonella enterica* Enteritidis (*S.*E.) assessed using a radial diffusion assay, as described in Methods. EW, total egg white proteins; HUB, heparin-unbound fraction of egg white; HB, heparin-bound fraction of egg white.

SDS-PAGE analysis of the samples recovered after heparin-Sepharose chromatography (Fig. 1a) revealed that egg white contains proteins with heparin-affinity including a 45–50 kDa major band (Fig. 1a, HB-EW). The protein profile of heparin-bound fraction (HB) is different from that of total egg white proteins (EW) and heparin-unbound fraction (HUB), demonstrating that low abundant egg white proteins have been enriched by heparin-affinity chromatography. It also suggests that heparin-binding proteins represent a minor part of total egg white proteins. Their concentration in egg white was estimated at least at 0.4 mg of proteins per mL of egg white, the total egg white proteins being usually around 100–120 mg/mL.

The lane corresponding to the heparin-binding fraction of egg white was cut into 15 sections (Fig. 1b) that were further analyzed by liquid chromatography coupled to tandem mass spectrometry (LC-MS/MS) for protein identification. In parallel, the complete fraction was analyzed in solution. Fourteen proteins were unambiguously identified by combining the in-gel and in-solution approaches (Table 1). All of these proteins are known components of the egg white, the vitelline membrane or the eggshell^8^. Some of them are recovered in many bands which reflect either degradation, multimerization or aggregation, as already reported for other egg proteins including ovalbumin or avidin^11^,^12^. Protein binding to heparin (negatively charged glycosaminoglycan) does not depend on protein pI as attested in Table 2 (pI range: 5.19–9.69) but rather implies a cluster of positively charged amino-acids^13^,^14^,^15^. Nine of the 14 chicken proteins identified or their mammalian homologs have been already identified as heparin-binding proteins (Table 2). The heparin-binding property of four of the remaining proteins needs further confirmation as ovalbumin, ovotransferrin, ovomucin and alpha-2-macroglobulin are major components of egg white, and are mainly recovered in the unbound fraction of egg white. Their presence in the fraction bound to heparin is likely to be fortuitous. Interestingly enough, the antibacterial activity of six of the proteins of this list or their mammalian counterpart have already been reported to be antibacterial: AvBD11^16^, OVAX^17^, lysozyme^18^, ovotransferrin^19^, avidin^20^, pleiotrophin^5^, and TENP^21^, which validates the initial strategy. An antibacterial activity against *Listeria monocytogenes* was observed in the diluted raw egg white and in both heparin-unbound and heparin-bound fractions to heparin, at 500 μg/mL (Fig. 1c). In contrast, we observed a slight activity of the heparin-bound fraction of egg white against *Salmonella enterica* Enteritidis whereas raw egg white and the unbound fraction were not active at 500 μg/mL.

### Purification and activity of antimicrobial candidates

**Figure 2:**
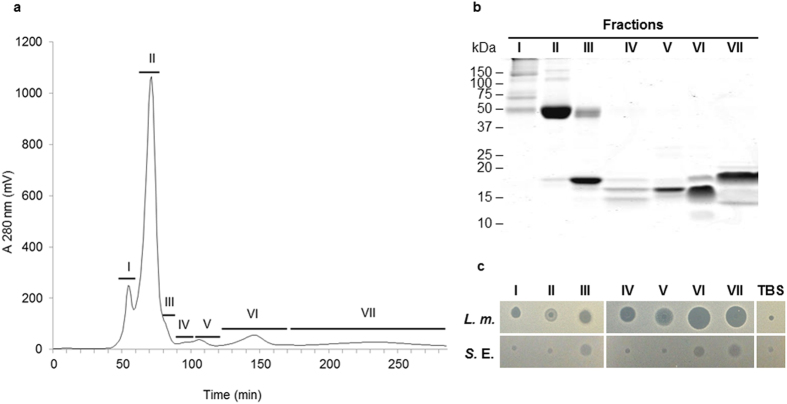
Analysis of gel filtration fractions containing heparin-binding proteins. (**a**) Chromatographic profile of heparin-binding proteins enriched fraction injected onto Hiprep 16/60 Sephacryl S-100 High Resolution. Seven fractions (I, II, III, IV, V, VI, VII) were collected and further concentrated onto a Ultracel-3 K centrifuge filter unit. (**b**) Electrophoretic profile of protein fractions I, II, III, IV, V, VI and VII (2 μg) by SDS-PAGE under non-reducing conditions. (**c**) Antibacterial activity of fractions I, II, III, IV, V, VI and VII (5 μL at 500 μg/mL for fractions I, II, III, V, VI and 5 μL at 150 μg/mL and 290 μg/mL for fractions IV and VII, respectively) against *Listeria monocytogenes* (*L.m.*) and *Salmonella enterica* Enteritidis (*S.*E.) assessed using a radial diffusion assay, as described in Methods.

The heparin-binding fraction of egg white was further separated by gel filtration chromatography (Fig. 2a). The various peaks were concentrated and analyzed for their antimicrobial activity and protein composition. Seven different peaks were obtained showing various electrophoretic profiles as assessed by SDS-PAGE (Fig. 2b). The seven fractions collected from gel filtration after heparin-affinity chromatography, were shown to be active against *Listeria monocytogenes* (Fig. 2c). Only three (Fractions III, VI and VII) of the seven fractions from gel filtration were shown to exhibit activity against *Salmonella enterica* Enteritidis (Fig. 2c). The seven fractions resulted from gel filtration were further analyzed for their protein composition by mass spectrometry (Table S1). The major results of this analysis are reported in the last column of Table 1. Ovalbumin-related protein X (OVAX) (gi|510032768) is the major component of fraction II (emPAI = 548) and is also recovered in fractions I and III. It corresponds to the large SDS-PAGE band of about 45–50 kDa in fraction II (Fig. 2b). Pleiotrophin (gi|444741724) is the second main constituent of Fraction III (emPAI = 19.36) and is also present to a lesser extent in the fractions II and VII. Vitelline Membrane Outer Layer Protein I (VMO-1) (gi|576329) is the most prominent protein of fractions V (emPAI = 2,336) and IV (emPAI = 494) and also appears in the fraction III. Gallinacin 11 precursor (AvBD11) (gi|49169808) is the major protein of fraction VI (emPAI = 1492) and is also present in fractions V and VII. PREDICTED: beta-microseminoprotein-like (gi|513191195) is the most concentrated protein composing fraction VII (emPAI = 1160). It was also found in fractions IV, V and VI. In addition to these five major components, this second approach based on heparin chromatography followed by gel filtration allowed the identification of six additional significant proteins that were not revealed in the first in-gel and in-solution proteomic analyses (supplemental file 1): Golgi apparatus protein 1 precursor (gi|45382795, Fraction I), PREDICTED: WAP four-disulfide core domain protein 3 isoform X5 (gi|513218625, Fraction II), peptidyl-prolyl cis-trans isomerase B (gi|45382027, Fractions III and IV), retinoic acid receptor responder protein 2 precursor (gi|477507238, Fraction IV), ovocleidin-17 (gi|32699622, essentially in fractions V and VI) and metalloproteinase inhibitor 3 (gi|45382757, Fractions V and VII). All these proteins have the potential *a priori* to bind heparin and to have antibacterial activity.

**Figure 3:**
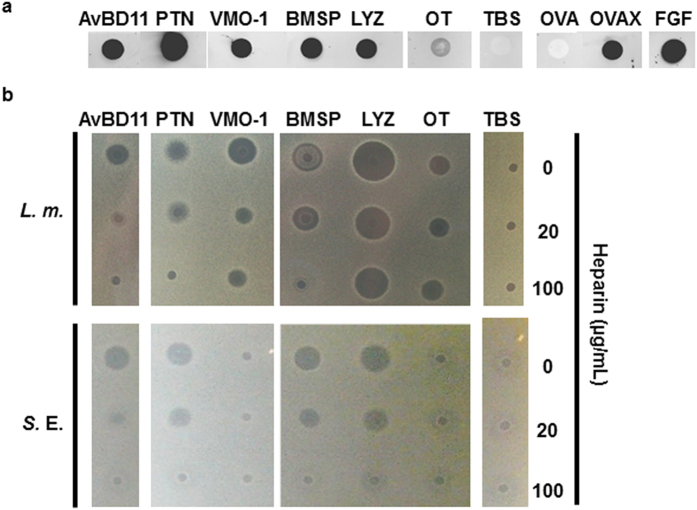
Effect of heparin on the antibacterial properties of purified heparin-binding candidates. (**a**) The heparin affinity of purified proteins was assessed by dot-blot using biotinylated-heparin, as described in Methods. FGF2 and OVAX were used as positive controls and OVA and TBS as negative controls. (**b**) The antibacterial activity of purified proteins (5 μL at 100 μg/mL) pre-incubated with heparin at 0, 20 and 100 μg/mL was assessed against *Listeria monocytogenes* (*L.m.*) and *Salmonella enterica* Enteritidis (*S.*E.) using a radial diffusion assay, as described in Methods. AvBD11 and TBS were used as positive and negative controls, respectively. AvBD11, avian beta-defensin 11; PTN, pleiotrophin; VMO-1, vitelline membrane outer layer protein 1; BMSP, beta-microseminoprotein-like; LYZ, lysozyme; OT, ovotransferrin; OVAX, ovalbumin-related protein X; OVA, ovalbumin; FGF, fibroblast growth factor 2; TBS, 50 mM Tris-HCl, 150 mM NaCl, pH 7.4.

To further characterize these two expected properties, we focused on the major proteins identified in this approach: AvBD11, that is also used as a positive control for antibacterial assays^16^, pleiotrophin, VMO-1 and beta-microseminoprotein-like as new candidates, lysozyme, ovalbumin and ovotransferrin as potential by-products of purifications. OVAX was previously demonstrated to exhibit antibacterial and heparin-binding properties^17^. We first investigated the heparin affinity of these candidates to determine whether the interaction of the proteins with heparin is direct or not and to better define the relevance of some of the major proteins of egg white in the heparin-bound fraction. In that purpose, these candidates were purified and assessed for heparin affinity by dot-blot with biotinylated heparin (Fig. 3a). Results showed that OVAX and AvBD11 bind heparin as well as the three new candidates (pleiotrophin, VMO-1, beta-microseminoprotein-like). Lysozyme was also detected to bind heparin which confirmed previous studies^22^, whereas ovalbumin and ovotransferrin were not or only at a very low level. These results suggest that these two highly abundant proteins were likely recovered in the heparin-bound fraction due to their interaction with other heparin-binding proteins (or with the Sepharose matrix beads) and that they do not have a direct affinity to this glycosaminoglycan. To prove the concept of the antibacterial activity of heparin-binding proteins, the purified proteins were investigated for their activity against *Listeria monocytogenes* and *Salmonella enterica* Enteritidis (Fig. 3b), two foodborne pathogens that were previously shown to be susceptible to AvBD11^16^ and OVAX^17^. Lysozyme, pleiotrophin, AvBD11 and beta-microseminoprotein-like were active against both strains at 100 μg/mL. VMO-1 and ovotransferrin were active at 100 μg/mL against *Listeria monocytogenes* but not *Salmonella enterica* Enteritidis (Fig. 3b) and ovalbumin exhibited no activity, regardless of the strain (not shown). The antibacterial activities of pleiotrophin, AvBD11 and beta-microseminoprotein-like against *Salmonella enterica* Enteritidis and *Listeria monocytogenes* were completely abolished in the presence of 100 μg/mL heparin, which suggests that the heparin-binding site is directly responsible for their respective antibacterial activity. Similar results were obtained with the anti-*Salmonella* activity of lysozyme. In contrast, the anti-*Listeria* activities of lysozyme and VMO-I were only partly reduced in the presence of heparin. This observation hypothesizes that the anti-*Listeria* effect of lysozyme and VMO-1 do not solely rely on the heparin-binding site(s).

## Discussion

Heparin-binding proteins are present in most living organisms and have a role in a variety of physiological functions including angiogenesis (pleiotrophin^23^) cell proliferation and migration (growth factors^24^), coagulation (antithrombin^25^), male fertility^26^ and innate immunity (cathelicidin, chemokines^27^; cathepsin G, elastase^28^). The heparin-binding domain is characterized by a stretch of positively charged amino-acids and would act similarly to cationic antimicrobial peptides, by interacting with the negatively charged bacterial surface such as lipopolysaccharide or lipoteichoic acid, which leads to the disruption of the bacterial plasma membrane^4^,^5^,^29^. In this context, we investigated the presence of heparin-binding proteins in the egg white (fluid known for its potent antibacterial activity), using heparin-affinity chromatography. Mass spectrometry analyses combined to antibacterial tests allowed us to identify several new antibacterial proteins in the chicken egg. The twenty proteins detected in the heparin-bound fraction are described and discussed in the next paragraphs.

### Heparin binding-proteins with known antibacterial activities

Avidin was predicted to display antimicrobial activity through its high affinity for biotin, an essential vitamin for some bacterial strains^30^. The fact that it binds to heparin-Sepharose suggests that it might have an additional antimicrobial role by interacting with negatively charged surfaces such as bacterial lipopolysaccharide, lipoteichoic acid or peptidoglycan. The antimicrobial properties of **lysozyme** reside mainly in its ability to hydrolyze bacterial peptidoglycans of Gram-positive bacteria^31^. Some have suggested that lysozyme bears antimicrobial activity that is independent of its enzymatic activity^32^. It has been previously shown to have antimicrobial activity against *L. monocytogenes*^33^. These two observations corroborate our results as heparin has only a small effect on the anti-*Listeria* activity of lysozyme. Indeed, the muramidase activity of lysozyme probably drives the major effect of this hydrolase against Gram-positive bacteria. In contrast, the anti-*Salmonella* activity of lysozyme is likely due the heparin-binding site since it is abolished when the site is occupied by heparin. It is noteworthy that lysozyme has only a weak affinity for heparin since it is mainly recovered in the unbound fraction of heparin-Sepharose (Fig. 1a, HUB-EW, 14 kDa band), which was also confirmed by Western-blot analysis (data not shown). Protein TENP (Transiently Expressed in Neural Precursor) is a protein possessing strong homology with members of the bactericidal/permeability increasing protein family (BPI)^34^. In emu egg, protein TENP is a protein of high abundance (it accounts for about 16% of total proteins in emu egg white) whereas it is relatively low concentrated in chicken egg white (0.1−0.5% of chicken egg white total protein content)^21^,^35^. Protein TENP in emu was shown to exhibit antibacterial activity against Gram-positive bacteria^21^. Considering the high sequence identity between emu and chicken forms (58%), chicken protein TENP is expected to be an antibacterial agent. Using this approach of heparin-chromatography, we also recently identified two egg antimicrobial heparin-binding proteins: AvBD11^16^ and OVAX^17^. **Avian beta-defensin 11** (AvBD11) belongs to the defensin family that encompasses natural antibiotic peptides. These peptides exert a broad-spectrum activity against a wide range of microbes and play a major role in innate immunity of many organisms^2^. AvBD11 has been identified for the first time in egg white by Mann *et al*.^8^ and purified by our group from egg vitelline membrane using heparin-Sepharose as the first step of purification^16^. This peptide exhibits a broad-spectrum activity as revealed by radial diffusion assays^16^. Its activity against both *L. monocytogenes* and *Salmonella enterica* Enteritidis strains was confirmed in the present study. We demonstrate here that AvBD11 directly binds to heparin and we showed that we could abolish its antibacterial activity by pre-incubating the peptide with heparin, which suggests that the heparin-binding domain is involved in its antibacterial property. Similarly, OVAX was previously shown to display antibacterial activity as opposed to its related protein, ovalbumin^17^. Its activity was also dependent on its heparin-binding domain^17^.

### New antimicrobial proteins in chicken egg

**Figure 4:**
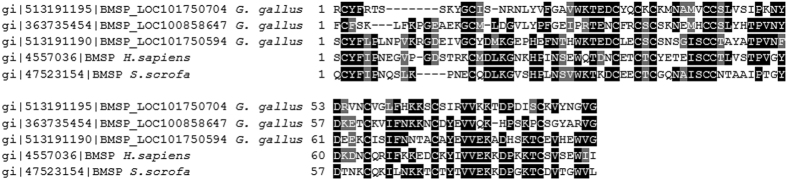
Protein sequence alignment of mature beta-microseminoproteins from chicken, human and boar. The following sequences were retrieved from the NCBI protein database: PREDICTED: beta-microseminoprotein-like [Gallus gallus] (GI:513191195), PREDICTED: beta-microseminoprotein-like isoform X1 [Gallus gallus] (GI:363735454), PREDICTED: beta-microseminoprotein A1-like [Gallus gallus] (GI:513191190), beta-microseminoprotein isoform a precursor [Homo sapiens] (GI:4557036) and beta-microseminoprotein precursor [Sus scrofa] (GI:47523154). Mature sequences were determined using SignalP 4.1 Server (http://www.cbs.dtu.dk/services/SignalP/). The alignment of mature sequences was performed using T-Coffee (http://www.ebi.ac.uk/Tools/msa/tcoffee/) with BLOSUM matrix and formatted with BOXSHADE (http://www.ch.embnet.org/software/BOX_form.html). Identical and similar residues are indicated in black boxes and grey boxes, respectively. BMSP, beta-microseminoprotein.

**Pleiotrophin** is a heparin-binding growth factor known for its mitogenic and angiogenic properties^36^,^37^ and the human homolog has recently been reported to exhibit potent bactericidal activity^5^. The chicken related protein has never been explored for such activities but considering the high sequence identity between chicken pleiotrophin and human pleiotrophin (93% sequence identity between respective mature forms), we suspect that the domains responsible for the antibacterial activity of the human pleiotrophin remain the same in its chicken counterpart. **Vitelline membrane outer layer protein 1** (VMO-1) is a major component of the outer vitelline membrane of hen egg together with ovomucin, lysozyme and AvBD11, and is also recovered in egg white^8^,^12^. However, its exact function in the egg is still unknown. The tridimensional structure of VMO-1 is organized into a singular structural motif called beta-prism, also found in the domain II of the insecticidal delta-endotoxin (a pore-forming toxin produced by *Bacillus thuringiensis*) and in the plant jacalin-like lectin domain^38^,^39^. Beta-prism motifs are thought to interact with carbohydrates. Delta-endotoxin domain II is involved in membrane receptor recognition and jacalin-like lectin domains are known to bind mannose. Structural studies showed that a cleft present in VMO-1 can accommodate carbohydrates^38^,^40^. Glycan synthetic activity and anti-hemagglutination properties were demonstrated for VMO-1^38^,^41^. VMO-1 is known to interact with the highly glycosylated protein ovomucin, the major structural component of the outer vitelline membrane, and it likely participates in membrane integrity^42^. No lytic activity similar to that of lysozyme was observed for VMO-1^41^. However, in this article, we report for the first time the ability of chicken VMO-1 to bind heparin and to inhibit *Listeria monocytogenes*. No antibacterial activity against *Staphylococcus aureus* and *Escherichia coli* was observed for human his-VMO-1^43^, which has 52.2% sequence identity with the chicken homolog. Interestingly, like lysozyme, the anti-*Listeria* activity of chicken VMO-1 is only partly abolished in the presence of heparin, which implies that it might exist a complementary mechanism by which VMO-1 can destroy bacteria and/or prevent its proliferation. Further study will focus on the mechanisms of action of VMO-1 on bacteria to better appreciate its functional domains. **Predicted: beta-microseminoprotein-like/LOC101750704** (gi:513191195) is a protein which protein sequence has been predicted by automated computational analysis by National Center for Biotechnology Information (NCBI). It is derived from a genomic sequence (NW_003763812.1) annotated using gene prediction method (Gnomon). Results from mass spectrometry analysis confirmed its presence, as 11 exclusive unique peptides were identified, covering 78.2% of the mature sequence (data not shown). Two different beta-microseminoprotein-related proteins (LOC101750594, LOC100858647) were previously identified in the eggshell^44^. In the present study, we identified beta-microseminoprotein-related protein (beta-microseminoprotein-like/LOC101750704) in the egg white for the first time. This protein has also recently been detected by mass spectrometry in the eggshell membrane proteome (UPI000350608B/XP_004942176.1^45^). All these three beta-microseminoprotein-related proteins are coded by three different clustered genes in chicken chromosome 6 (GeneID: 100858647, 101750594, and 101750704). The human homolog was shown to play a role in male fertility as a component of seminal plasma^26^. This protein is also present in nasal secretions and a role in mucosal innate immunity was previously suggested^46^,^47^. Human beta-microseminoprotein possesses a potent fungicidal activity against *Candida albicans* whereas no antibacterial activity was observed against *E. coli, S. agalactiae, S. pyogenes, S. aureus and E. faecalis*^48^. The boar homolog was described to bind heparin^49^. The chicken protein (LOC101750704) is about 9.9 kDa in its mature form (87 amino-acids) and contains 5 disulfide bonds, by similarity with the tridimensional structure of the dimer of the human homolog^50^. The sequence alignment of this protein with that of human and boar homologs and the two other chicken related proteins showed some marked differences (Fig. 4). The high cationicity of the chicken beta-microseminoprotein-like LOC101750704 (pI = 9.3) as compared with the two other chicken beta-microseminoprotein-related proteins LOC101750594 and LOC100858647 (4.67 and 8.32, respectively) and with human and boar beta-microseminoproteins (5.4 and 8.07, respectively), might be associated with some functional divergences. The fact that chicken beta-microseminoprotein is active against the two bacterial strains tested together with its high cationicity suggests that it might participate in the egg defense. In the future, it will be very interesting to evaluate the tissue specificity of all these chicken beta-microseminoproteins and their concentration in the egg, to compare the activity of all three homologs, to enlarge the antibacterial screening to other microbes in order to have a better idea of their respective antimicrobial potential.

### Other less abundant candidates

Mass spectrometry analyses of the seven fractions resulting from gel filtration allowed us to identify other candidates. The direct binding of heparin to these proteins to heparin could not be confirmed in the present study as we did not have the corresponding purified molecules. But, interestingly, the integration of the data from literature reveals that these less abundant candidates (chosen amongst the five more abundant proteins composing each fraction) were either already suggested to display antibacterial activity or at least do have a link with innate immunity. **Ovocleidin-17** was initially described as a major component of the calcified eggshell. It is a 142 amino-acids phosphorylated protein with a C-type lectin domain^51^, displaying bactericidal activity against *Bacillus subtilis*, *Staphylococcus aureus* and *Pseudomonas aeruginosa*^52^. **Metalloproteinase inhibitor 3 precursor** (TIMP3) is supposed to inhibit some metalloproteases, by similarity with its homologs. Its targeted protease in egg could be matrix metalloproteinase 2, which activity is regulated during embryogenesis^53^. A role for TIMP3 as an antibacterial agent has not been investigated yet.

**Retinoic acid receptor responder protein 2** is a new adipokin with has shown to exhibit various functions in reproduction^54^, in lipid and carbohydrate metabolism^55^ but also in innate immunity^56^. Indeed, it seems to act as a chemotactic factor for leukocytes, macrophages and natural killer cells^57^. Its direct role to counteract bacterial infections has not been demonstrated but it might indirectly play a role in response to microbial attacks by recruiting inflammatory cells on the site of infection. Its presence in freshly laid egg white might reflect some inflammation status of the upper part of the oviduct during egg formation. **Golgi apparatus protein 1** also called E-selectin ligand 1 is a high molecular weight protein, which is likely to play a role in inflammation processes by allowing the migration of neutrophils^58^. It is noteworthy that the expression of this protein is early stimulated upon lipopolysaccharide infection^59^, which underlines its role in the cascade of events underlying the host immune response. **Peptidyl prolyl isomerase B** (Cyclophilin B) is known to accelerate the folding of proteins and would therefore have a role in the acquisition of functional conformation of proteins^60^. It has been shown in other species to be involved in inflammatory events. It can induce chemotaxis in human neutrophils and T lymphocytes and requires local glycosaminoglycans for optimal activity^61^. It binds to the immunosuppressor agent cyclosporin A and was demonstrated to be important during viral infection^62^. **Clusterin** is also a potent ubiquitous extracellular chaperone that inhibits protein aggregation and precipitation caused by physical or oxidative stresses^63^. To our knowledge, the role of this chaperone protein as an antibacterial agent has never been reported. Finally, we report the presence of two additional predicted egg white proteins, **PREDICTED: mucin-5B isoform X1** and **PREDICTED: WAP four-disulfide core domain protein 3 isoform**, which both presumably bind heparin. The functional characterization of these two proteins has not been initiated yet. But, considering that mucins play a critical role in the mucosal immune function of the chicken respiratory and gut tracts^64^,^65^ and are responsible for the differential effector and regulatory responses against microbial infections^66^, and that WAP domains proteins are also involved in mucosal immunity^67^,^68^, it would not be surprising that these two proteins would also participate in the protection of the egg against pathogenic microorganisms.

To conclude, this work supports the fact that egg white is an important source of antibacterial proteins and peptides. And, knowing that some heparin-binding proteins and peptides including synthetic peptides derived from the heparin-binding domain(s) of heparin-binding proteins, exhibit activity against *Candida albicans*^4^,^69^,^70^, heparin-binding molecules identified in this study are likely to display similar antimicotic activities. Such molecules would be of great interest as new anti-infectious drugs as they could serve as templates to design multifaceted agents bearing a large range of antimicrobial activities. Additionally, we demonstrated that searching heparin-binding proteins in biological milieus bring essential information about both antimicrobial active proteins and essential immune effectors, giving an instantaneous snapshot of many actors of innate immunity. This strategy could be transposed to many other complex biological samples including human fluids (saliva, urine, amniotic fluid, etc.) to investigate innate immunity in physiological and pathological situations.

Altogether these data provide new insights and new tools for the scientific community to explore immunity, but also for industrials that are interested in the identification and valorization of natural antimicrobial proteins and peptides to be used as pharmaceuticals.

## Methods

### Materials

Heparin-Sepharose 6 Fast Flow Affinity Chromatography Media and nitrocellulose blotting membrane (0.2 μm) were obtained from GE Healthcare (Velizy-Villacoublay, France). Streptavidin Alexa Fluor 680 conjugate was purchased from Molecular Probes (Fisher Scientific, Saint-Aubin France). Enoxaparin, heparin from porcine intestinal mucosa and bovine serum albumin were from Sigma-Aldrich (Sigma-Aldrich, Saint Quentin Fallavier, France). All other chemicals were of analytical grade.

### Purification of egg white heparin-binding proteins

Egg whites were collected from freshly laid eggs (Isa-Hendrix, St Brieuc, France) and were homogenized using an Ultra-Turrax homogenizer (T18 basic ULTRA-TURRAX, IKA-Werke, Staufen, Germany), sampled and kept frozen until further use.

Heparin-Sepharose chromatography using the batch method was performed according to manufacturer’s instructions. Briefly, egg white (see above) was diluted 1:1 in 50 mM Tris-HCl, 150 mM NaCl, pH 7.4 and incubated with heparin-Sepharose beads (10:1, v/v) overnight at 4 °C under constant but slow agitation. The next day, the beads were washed extensively with 50 mM Tris-HCl, 150 mM NaCl, pH 7.4 until the absorbance at 220 nm reached zero, and were loaded onto a polypropylene column (QIAGEN, Courtaboeuf, France). Elution of bound proteins was achieved with 50 mM Tris-HCl, 1 M NaCl, pH 7.4. Eluted fractions were concentrated (Ultracel-3K, Merck Millipore, Molsheim, France) and injected on a gel filtration column (Hiprep 16/60 Sephacryl S-100 High Resolution, GE Healthcare Life Sciences, Velizy-Villacoublay, France) using 50 mM Tris-HCl, 300 mM NaCl, pH 7.4, as the mobile phase. Proteins composing each fraction and purified proteins were analyzed by 12.5% SDS-PAGE under non-reducing conditions followed by Coomassie Blue staining. Major peaks were collected and concentrated by ultracentrifugation, as described above. Beta-microseminoprotein-like (gi|513191195) was further purified by gel filtration. Vitelline membrane outer layer protein 1 (gi|268370086) and pleiotrophin (gi|444741724) were purified from the salt-soluble part of the vitelline membrane, successively by heparin-affinity chromatography, gel filtration and reverse-phase chromatography as described previously for AvBD11^16^. Purified AvBD11 and OVAX were obtained as previously described^16^,^17^. The protein concentrations of OVAX (Ext. coefficient E1% = 9.11), AvBD11 (Ext. coefficient E1% = 15.86), VMO-1 (Ext. coefficient E1% = 21.13), pleiotrophin (Ext. coefficient E1% = 15.87) and beta-microseminoprotein-like (Ext. coefficient E1% = 15.18) were measured using absorbance at 280 nm and their respective E1% values (Nanodrop, ND-1000 Spectrophotometer, Wilmington, USA). The purity of purified proteins was systematically verified by SDS-PAGE and mass spectrometry.

### Study of the heparin affinity of purified proteins by dot-blot

Direct binding of heparin-binding candidates to heparin was assessed by dot-blot using manufacturer’s instructions (Bio-Dot SF Microfiltration Apparatus, Bio-Rad, Marnes-la-Coquette, France). Low molecular weight (LMW) heparin (enoxaparin) was labeled with biotin as described by Gerlza *et al*.^71^. Briefly, CH3BNNa (200 mg) was dissolved in 2 M NH4Cl (2 mL) and pipetted into dissolved enoxaparin (2 mL at 5 mg/mL in 2 M NH4Cl). The reaction was incubated for 48 h at 70 °C under shaking. Then, another 100 mg of CH3BNNA was added and incubated for additional 48 h at 70 °C. Dialysis against phosphate buffer saline (PBS) was performed using Float-A-Lyzer 0.5–1 kDa (Spectrum Labs, Rancho Dominguez, CA, USA). The sample volume was reduced to 2.5 mL using a SpeedVac concentrator and was filtrated through a 0.22 μ filter. The biotinylation reaction was performed on ice for 2 h with 7.5 mg of biotin (EZ-Link Sulfo-NHS-LC-Biotin, Thermo Fisher Scientific, Courtaboeuf, France). To eliminate the unbound biotin, the reaction mixture was desalted in a Float-A-Lyzer 0.5–1 kDa against demineralized water and lyophilized. Resulting samples were kept at 4 °C until further use.

Purified heparin-binding proteins (1 μg) diluted in 200 μL of 50 mM Tris-HCl, 150 mM NaCl, pH 7.4 were spotted onto a 0.22 μm nitrocellulose membrane and washed twice with 50 mM Tris-HCl, 150 mM NaCl, pH 7.4 using the 96-well Bio-Dot apparatus (Bio-Rad, Marnes-la-coquette, France). Membranes were blocked in 50 mM Tris-HCl, 150 mM NaCl, pH 7.4 containing 1% bovine serum albumin, overnight at 4 °C. Incubation with biotinylated heparin (100 μg/mL in 50 mM Tris-HCl, 150 mM NaCl, pH 7.4, 1% bovine serum albumin) was performed at room temperature during 1 h. The membrane was washed with 50 mM Tris-HCl, 150 mM NaCl, pH 7.4 and incubated with Streptavidin Alexa Fluor 680 conjugate (1/3000 in 50 mM Tris-HCl, 150 mM NaCl, pH 7.4, 1% bovine serum albumin) during 1 h at room temperature. Analysis of the membrane was realized using an infrared imaging system (Odyssey, LI-COR Biosciences Inc., Lincoln, NE, USA).

### Radial diffusion assay

Antibacterial tests were conducted using a radial diffusion assay as described by Lehrer^72^. Pathogenic bacterial strains, *Salmonella enterica* serovar Enteritidis ATCC 13076 (*S.* Enteritidis) and *Listeria monocytogenes* EGD strain (*L. monocytogenes)* were provided by the International Centre for Microbial Resources (CIRM) from the French National Institute for Agricultural Research (INRA, France). Pre-cultures of *S.* Enteritidis and *L. monocytogenes* were performed overnight in Trypticase Soy Broth (TSB, BD Biosciences) and in Brain Heart Infusion broth (BHI, BD Difco), respectively. This pre-culture was then used to inoculate a new culture broth (TSB or BHI) so that the mid-exponential phase was obtained after 3 to 4 hours of incubation depending on strains, with shaking at 37 °C.

Bacteria were centrifuged at 900 g for 10 min at 4 °C, washed twice with cold 10 mM sodium phosphate buffer (pH 7.4) and re-suspended in cold sodium phosphate buffer. Bacteria (7.5 × 10^6^ CFU) were mixed with 25 ml of previously autoclaved, warm 10 mM phosphate buffer containing 0.03% TSB medium, 1% (wt/vol) low-endosmosis agarose (Sigma-Aldrich, Saint Quentin Fallavier, France), and 0.02% Tween 20, knowing that 1 OD unit corresponds to 2.33 × 10^9^ CFU/mL and to 6.125 × 10^8^ CFU/mL for *L. monocytogenes* and *S.* Enteritidis, respectively. The assay was further processed as previously described^16^ using 5 μL of protein samples (at 500 μg/mL or 100 μg/mL in 50 mM Tris-HCl, 150 mM NaCl, pH 7.4) in each well. The effect of heparin on the activity of antibacterial proteins was assessed by incubating purified proteins (100 μg/mL) with heparin from porcine intestinal mucosa at 0, 20 and 100 μg/mL in 50 mM Tris-HCl, 150 mM NaCl, pH 7.4 before introduction into the well.

### Mass spectrometry analyses

Unbound fraction of heparin-Sepharose (250 μg) was diluted in Laemmli buffer under non-reducing conditions, separated by SDS-PAGE on a 4–20% gradient gel and further stained with Coomassie blue. The lane corresponding to heparin-binding proteins was cut into 15 sections for further analysis by mass spectrometry. Each section was rinsed with water and acetonitrile, then reduced with dithiothreitol, alkylated with iodoacetamide and incubated overnight at 37 °C in 25 mM NH_4_HCO_3_ with 12.5 μg/mL trypsin (Sequencing Grade, Roche, Paris) as described by Shevchenko *et al*.^73^. The tryptic peptides were extracted, dried, reconstituted with 0.1% formic acid, and sonicated for 10 min and sequenced by nanoscale liquid chromatography-tandem mass spectrometry (nanoLC-MS/MS) using CapLC system coupled to a hybrid quadrupole time-of-flight mass spectrometer (Q-Tof Ultima Global, Waters, Micromass, Manchester, UK) as previously described^74^. Data were processed using a ProteinLynx Global server 2.2 (Waters) and peak lists were exported in PKL format file. All PKL files were merged into MGF format file for a single database search. In order to identify the proteins, the precursor and corresponding ion fragment masses were matched automatically against a locally maintained copy of the non-redundant nr NCBI database (downloaded 8 July 2015) in the Chordata section (2958458 sequences) using Mascot v2.3 software (Matrix Science, London UK). Enzyme specificity was set to trypsin with 2 missed cleavages using carbamidomethylcysteine, oxidation of methionine and N-terminal protein acetylation as variable modifications. The mass tolerance was 0.2 Da for precursors and fragment ions. Dried samples were solubilized with 50 mM ammonium bicarbonate, reduced with 5 mM dithiothreitol, alkylated with 12.5 mM iodoacetamide and incubated overnight at 37 °C with trypsin using an enzyme/substrate at 1/40 (Sequencing Grade, Roche, Paris). Peptide mixtures acidified at 0.1% formic acid were directly analyzed by nanoLC-MS/MS using a LTQ Orbitrap Velos high resolution mass spectrometer (Thermo Fisher Scientific, Bremen, Germany) coupled to an Ultimate 3000 RSLC Ultra High Pressure Liquid Chromatographer (Dionex, Amsterdam, The Netherlands) as previously described. Raw data files were converted to MGF with Proteome Discoverer software (version 1.2; Thermo Fischer Scientific, San Jose, USA). Data were matched against the nrNCBI database using the parameters as described above, at the exception that the tolerance of the ions was set at 5 ppm for parent and 0.8 Da for fragment ion matches.

All Mascot results (in-gel and in-solution analyses) were incorporated in Scaffold 3 software (version 4.2, Proteome Software, Portland, USA). Peptide identifications were accepted if they could be established at greater than 95.0% probability as specified by the Peptide Prophet algorithm^75^. Protein identifications were accepted if they could be established at greater than 95.0% probability as specified by the Protein Prophet algorithm^76^ and contained at least two identified peptides. The abundance of identified proteins was estimated by calculating the emPAI^77^.

## Additional Information

**How to cite this article**: Guyot, N. *et al*. Proteomic analysis of egg white heparin-binding proteins: towards the identification of natural antibacterial molecules. *Sci. Rep.* **6**, 27974; doi: 10.1038/srep27974 (2016).

## Supplementary Material

Supplementary Information

## Figures and Tables

**Table 1:** Mass spectrometry analysis of heparin-binding proteins from egg white.

Identified Proteins (14)	Accession Number	Gene ID	MW (kDa)	in-gel identification					in-solution identification				Gel filtration fraction(s) containing the identified protein*
				emPAI	exclusive unique peptide count	Percent coverage	Total Spectrum count	Gel Section	emPAI	exclusive peptide count	Percent coverage	Total spectrum count	
Ovalbumin-related protein X	gi|448824824	420898	44	12.8	25	68	109	1,2,3,4,6,7,8, 9,10,11,15	1.3	7	0.2	198	**I**, **II**, **III**, IV, V, VI, VII
Avian beta-defensin 11	gi|49169808	414876	12	2.2	3	27	11	13,14	10	6	0.5	127	I, II, III, IV, V, **VI**, VII
Avidin	gi|112490098	396260	30	1.6	6	25	19	1,2,3,13	–	–	–	–	I, II, III
Lysozyme	gi|229157	396218	14	1.1	3	33	3	14	1.1	3	0.3	20	I, II, VI, VII
PREDICTED: beta-microseminoprotein-like	gi|513191195	101750704	12	0.8	2	17	2	12	1.4	3	0.3	22	I, II, III, IV, V, VI, **VII**
Vitelline membrane outer layer protein 1	gi|268370086	418974	20	0.4	2	16	3	13	1.1	4	0.3	35	III, **IV**, **V**, VI, VII
OvoglobulinG2/TENP	gi|385145523	395882	47	0.4	4	18	9	7,8	0.1	1	0	12	I, II
PREDICTED: alpha-2-macroglobulin-like 1	gi|513161014	418254	147	0.2	9	11	13	2,3,4	0.1	3	0	4	I, II
Ovalbumin	gi|129293	396058	43	0.2	2	10	2	7	–	–	–	–	I, II, III, IV
Olfactomedin-like protein 3	gi|90968644	419882	45	0.2	2	6.6	2	6	–	–	–	–	I, II, III, IV
Ovotransferrin	gi|83754919	396241	76	0.1	2	3.9	2	8	–	–	–	–	I, II
PREDICTED: mucin-5B	gi|363734560	395381	234	0.01	1	1	1	2	0.2	10	0.1	40	I, II, III, IV
Clusterin precursor	gi|45382467	395722	51	0.07	1	4	2	2,9	0.2	3	0.1	16	I, II
Pleiotrophin precursor	gi|444741724	418125	19	–	–	–	–	–	0.5	2	0.2	23	I, II, III, IV, V, VI, VII

MW, molecular weight

^*^According to the MS data of gel filtration fractions (see Fig. 2 and Table S1). Fractions in bold correspond to fractions where the protein was the most prominent protein, based on EmPAI values (Table S1).

**Table 2:** Characteristics of identified proteins.

Identified Proteins (14)	Gene ID	pI	Heparin-affinity	Antimicrobial activity	Egg localization
Ovalbumin-related protein X	420898	6.29	17	17	ES, EW, VM, EY
Avian beta-defensin 11	414876	8.78	16	16	ES, EW, VM
Avidin	396260	9.69	78	20	ES, EW, VM, EY
Lysozyme	396218	9.32	22	18	ES, EW, VM, EY
PREDICTED: beta-microseminoprotein-like	101750704	9.23	Boar beta-microseminoprotein^49^	Human beta-microseminoprotein^48^	EW (this study), ES
Vitelline membrane outer layer protein 1	418974	8.85	–	–	ES, EW, VM, EY
OvoglobulinG2/TENP	395882	5.77	Human recombinant rBPI23^79^	Emu TENP^21^	ES, EW, VM, EY
PREDICTED: alpha-2-macroglobulin-like 1	418254	8.52	–	–	ES, EW, VM
Ovalbumin	396058	5.19	–	–	ES, EW, VM, EY
Olfactomedin-like protein 3	419882	5.56	Mouse olfactomedin^80^	–	EW, VM
Ovotransferrin	396241	6.91	–	19	ES, EW, VM, EY
PREDICTED: mucin-5B/ovomucin	395381	5.35	–	–	ES, EW, VM
Clusterin	395722	5.47	Human clusterin^81^	–	ES, EW, VM, EY
Pleiotrophin	418125	9.63	Human pleiotrophin^82^	Human pleiotrophin^5^	ES, EW

–, lacking information; ES, eggshell; EW, egg white; EY, egg yolk; VM, vitelline membrane.
